# The clinical significance of remnant thyroid tissue in thyroidectomized differentiated thyroid cancer patients on ^131^I-SPECT/CT

**DOI:** 10.1186/s12880-021-00612-5

**Published:** 2021-05-08

**Authors:** Feng Wang, Hui Nie, Wei Li, Rusen Zhang, Wen Li

**Affiliations:** 1 Department of Nuclear Medicine, Affiliated Cancer Hospital & Institute of Guangzhou Medical University, Guangzhou, 510095 Guangdong Province China; 2 Department of Health Care, Affiliated Cancer Hospital & Institute of Guangzhou Medical University, Guangzhou, 510095 Guangdong Province China

**Keywords:** Iodine-131, SPECT/CT, Differentiated thyroid cancer, Postoperative remnant, Thyroidectomy, Trachea cartilage, Thyroid cartilage

## Abstract

**Background:**

To explore the ^131^I-SPECT/CT characteristics of remnant thyroid tissue (RTT) in differentiated thyroid cancer (DTC), further assess the risk factors and clinical significance.

**Methods:**

52 DTC patients after total thyroidectomy had undergone neck ^131^I-SPECT/CT before ^131^I ablation. The diagnosis of RTT was based on SPECT/CT and follow-up at least 3 months. The anatomic locations and features of SPECT/CT of RTT were assessed by reviewers. The risk factors of RTT with CT positive were analyzed by the chi-square test.

**Results:**

A total of 80 lesions of RTT were diagnosed in this study, most of them were mainly located in the regions adjacent to trachea cartilage (37/80) or lamina of thyroid cartilage (17/80). On SPECT/CT of RTT, low, moderate and high uptake were respectively noted in 10, 24 and 46 lesions, definite positive, suspected positive and negative CT findings were respectively noted in 10, 21 and 49. The RTT lesions with definite positive CT findings were mainly located adjacent to lamina of thyroid cartilage (5/10). Primary thyroid tumor (*P* = 0.029) and T stage (*P* = 0.000) were the effective risk factors of CT positive RTT.

**Conclusions:**

RTT has certain characteristic distribution and appearances on SPECT/CT. Most of RTT with definite CT abnormalities located adjacent to lamina of thyroid cartilage, which suggest surgeons should strengthen the careful removal in this region, especially primary thyroid tumor involving bilateral and T4 stage. This study can provide a certain value for the improvement of thyroidectomy quality in DTC patients.

## Background

Differentiated thyroid cancer (DTC) is one of the most common endocrine malignant tumor, including papillary thyroid carcinoma (PTC) and follicular thyroid carcinoma (FTC), which has the gradual increase rates of incidence and death in recent years [1–3]. Total thyroidectomy, radioactive iodine-131(^131^I) ablation, and thyroid stimulating hormone(TSH) suppression are well established treatments for DTC [4]. Complete surgical resection will be beneficial to decrease the risk of recurrence and mortality, and improve the prognosis of DTC patients [5].

However, due to the complex anatomy around thyroid, the surgical operation can easily cause the injury to important tissues such as recurrent laryngeal nerve (RLN) and parathyroid gland, which may lead to serious complications. In order to avoiding these complications, it is generally difficult for thyroidectomy to avoid the occurrence of remnant thyroid tissue (RTT), even for very skilled surgeons. RTT can cause the recurrence or metastasis of DTC, and badly influence the clinical prognosis [6]. Therefore, in order to maximize the benefits of patients, it is still the responsibility of every surgeon to carefully remove the thyroid tissue to minimize RTT. But, the effect of reducing RTT and improving the quality of surgery has been very little in recent years. It may be associated with the fact that the distribution characteristics and risk factors of RTT have not been systematically summarized, which makes careful surgical clearance lack of a clear guidance.

RTT should be routinely detected and evaluated after thyroid surgery, which can be used to guide the individualized treatment of ^131^I ablation in the next step. The comparison of diagnostic value of various methods for the detection of RTT can be seen in a large number of previous studies [7–10]. ^131^I-SPECT/CT (single photon emission computed tomography/ computed tomography) is recognized to be highly sensitive and specific, which has been widely used in clinical evaluation practice. However, the ^131^I-SPECT/CT features of RTT have not been reported in detail. Especially, when the RTT lesions were revealed the definite CT abnormalities on ^131^I-SPECT/CT, its clinical significance for surgeons remains to be further analyzed and researched.

Therefore, this study is aimed to explore the distribution and imaging characteristics of RTT in patients with DTC on ^131^I-SPECT/CT, to further analyze the risk factors, and to discuss its values for surgeons.

## Materials and methods

### Ethics statement

The present study was approved by the ethics committees of affiliated Cancer Hospital & Institute of Guangzhou Medical University. All patients provided written informed consent for their clinical information to be reviewed by us. And all methods were carried out in accordance with the approved guidelines.

### Patients

#### Inclusion criteria

Patients were included in this study if they (1) had a diagnosis and pathological confirmation of DTC at the affiliated Cancer hospital & Institute Guangzhou medical university between January 2020 and July 2020, (2) have been performed total thyroidectomy and cervical lymph node dissection, (3) have clear TNM staging according to the thyroid cancer staging criteria of AJCC (American Joint Committee on Cancer) 8th edition, (4) received the first radioiodine ^131^I therapy within 1–3 months after surgery, (5) have been performed ^131^I-WBS (whole body scan) and neck ^131^I-SPECT/CT within 1 week before radioiodine therapy.

#### Exclusion criteria

Patients were excluded from this study if they (1) underwent subtotal thyroidectomy, (2) lacked of information about the primary tumor, (3) had a synchronous malignant tumor of other site, (4) had not be followed up for more than 3 months.

Finally, a total of 52 patients with DTC were included in this study, 25 females and 27 males, aged range 11–67 years, median age 40 years. Among them, pathology confirmed the diagnosis of PTC in 50 cases and FTC in 2 cases.

### Surgery

All patients had received the surgical operations of total thyroidectomy and cervical lymph node dissection. There were three kinds of operation modes, mode 1 of “complete thyroidectomy + unilateral central neck lymph node dissection”, mode 2 of “complete thyroidectomy + bilateral central neck lymph node dissection” and mode 3 of “standard or modified radical operation”. All patients were determined the TNM staging according to the ACJJ 8th edition after surgery.

### WBS and SPECT/CT acquisition

All patients underwent scintigraphy scanning (WBS and SPECT/CT) within 3 months after surgery, using a SPECT/CT scanner (Discovery NM/CT 670 Pro, GE medical systems, Israel) with dual-headed gamma camera system, high-energy collimators and a 16-slice spiral scanning diagnostic CT. The WBS images were acquired 24–48 h after taking orally ^131^I (Atomic High-Tech Co. Ltd., Guangzhou, China) of 111–185 MBq, using a high-energy general purpose parallel holes collimator with 364 keV photopeak and 256 × 1024 matrix. Neck hybrid SPECT/CT scanning were routinely performed after WBS acquisition. CT scan was firstly performed and the acquisition parameters were as follows: tube voltage 140 kV, tube current 200 mA and matrices 512 × 512. After CT acquisition, the SPECT acquisition protocol was started, and the parameters were as follows: 128 × 128 matrix, 20% energy windows at 364 keV, 60° angular steps with a range of 180° per gamma camera head. JET stream workstation (Philips Medical Systems) was applied to obtain the fusion images of SPECT/CT.

### Image interpretation

The radionuclide images (WBS and SPECT/CT) were independently evaluated by 2 experienced nuclear medicine physicians with interpretation in consensus, using diagnostic software (Compass viewer H 4.0, Medivoly Technology Co. Ltd., Shanghai, China).

### Diagnosis of RTT

Reviewers were required to determine if there was abnormal ^131^I uptake foci on neck. The neck foci would be routinely diagnosed as RTT, if they were located in and near the thyroid bed, ruled out the diagnosis of metastatic lymph nodes, excluded the possibility of common ectopic thyroid areas such as thyroglossal duct and root of tongue, and observed the significant decrease of ^131^I radioactive concentration in the follow-up [11, 12].

### Locations of RTT

**Fig. 1:**
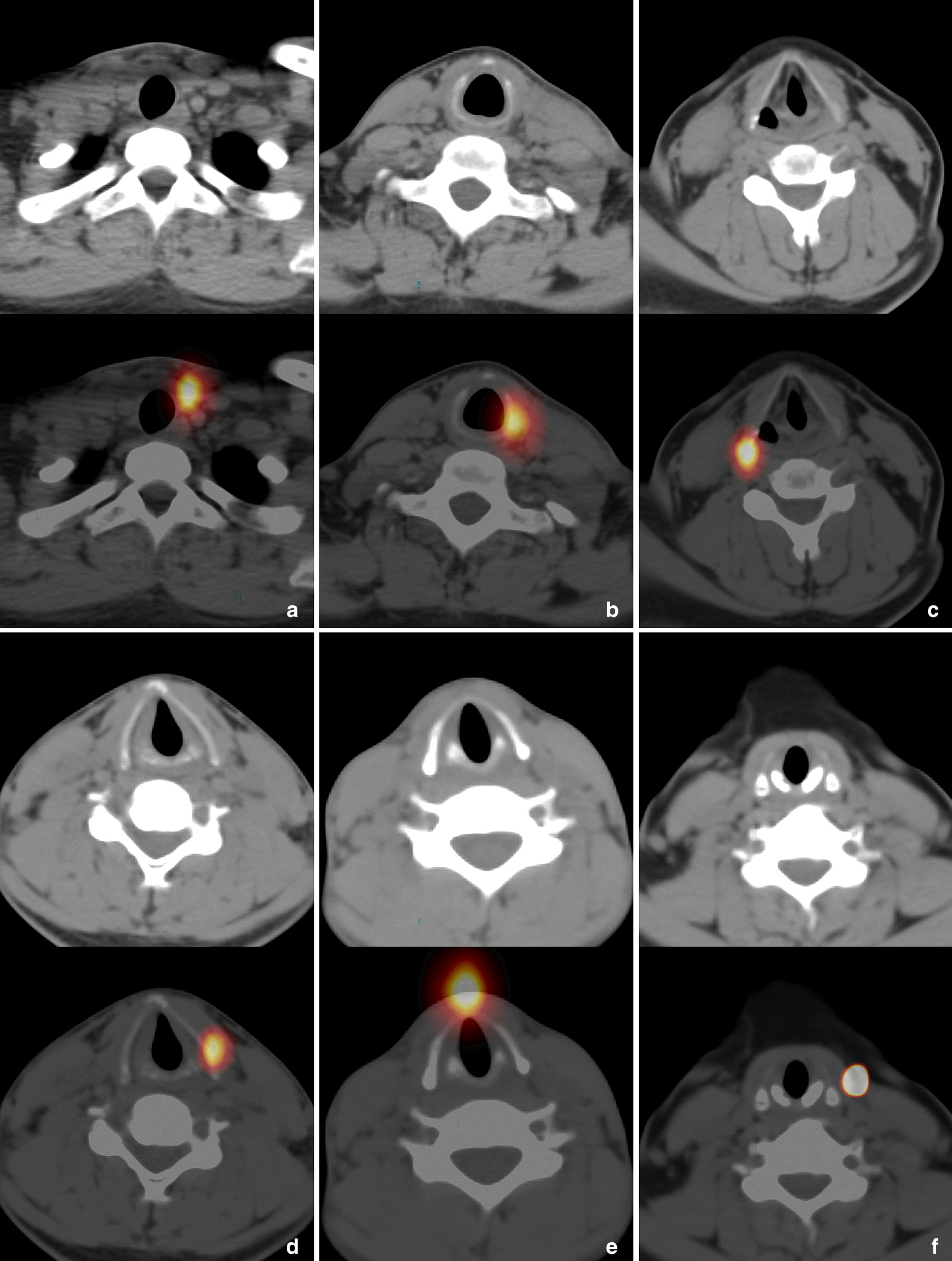
The regions (I–VI) of RTT. In cases of **a**–**f**, the RTT lesions with high-uptake tracer activity were located respectively in region I (adjacent to the left side of tracheal cartilage), region II (adjacent to the left side of cricoid cartilage), region III (adjacent to the right posterior border of thyroid cartilage), region IV (adjacent to the left thyroid lamina), region V (adjacent to the midline of anterior thyroid cartilage) and region VI (outside the left infrahyoid muscle)

The anatomic locations of RTT on neck SPECT/CT were recorded in detail. According to the distribution characteristics, the locations of RTT were divided into 6 regions (I–VI). The detailed definitions and example images of regions were shown in Table 1 and Fig. 1.

**Table 1:** The regional definition of RTT

Region	Definition
I	Adjacent to the tracheal cartilage
II	Adjacent to the cricoid cartilage
III	Adjacent to the posterior border of thyroid cartilage
IV	Adjacent to the thyroid lamina
V	Adjacent to the midline of anterior thyroid cartilage
VI	Other areas

### SPECT/CT findings of RTT

The features of SPECT/CT of RTT were assessed by reviewers. According the CT findings, the lesions of RTT were divided into three types, type I of definite positive findings (obvious soft tissue nodule), type II of suspected positive findings (blurry patchy shadow or soft tissue thickening), and type III of negative findings (no thicken soft tissue shadow and abnormal density). The tracer uptake level of RTT on SPECT were split into low-, moderate-, and high- uptake based on whether to be lower than, equal to or higher than those of the stomach.

### The clinical data of patients

The patients with RTT of the positive CT findings (type I or/ and II) were assigned to positive group, otherwise to negative group. According to the extent of invasion, the primary thyroid tumor can be divided into unilateral (one lobe and/ or isthmus) and bilateral (double lobes). In addition, all patients were collected data as follows: age (< 55 and ≥ 55) and gender (male and female), T stage (T1, T2 and T3–4), N stage (N0 and N1), M stage (M0 and M1), and pathology (PTC and FTC).

## Statistical analysis

The incidence rate of RTT after total thyroidectomy was calculated in DTC patients. The clinical data and imaging features of RTT were counted in detail. Categorical data are expressed as numbers and frequency (%). The chi-square test was used to analyze the effective risk factors of RTT with positive CT findings.

All data was analyzed by SPSS 23.0 for windows (SPSS Inc., Chicago, IL, USA) software. *P* value < 0.05 were considered statistically significant.

## Results

### All patients

**Fig. 2:**
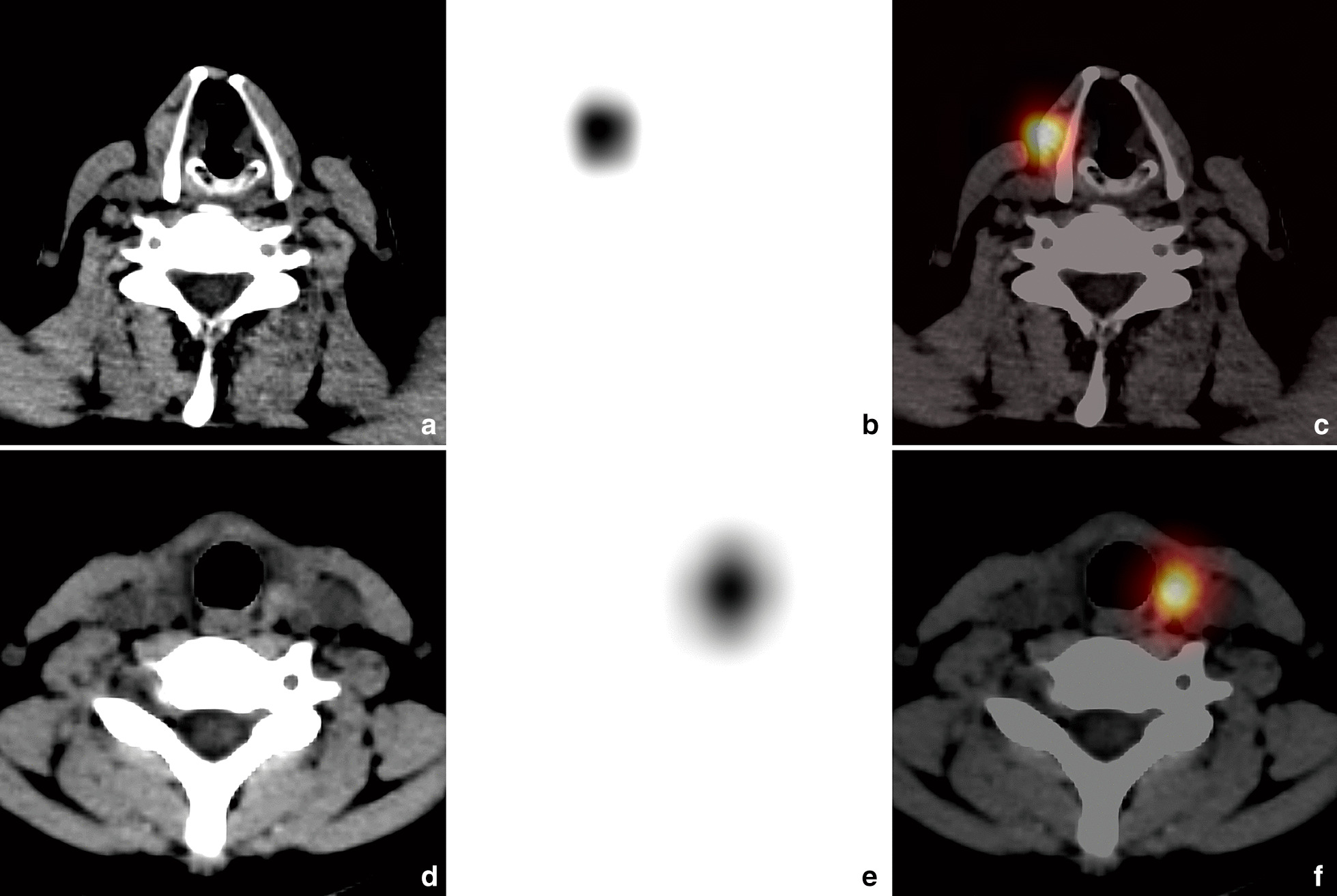
RTT lesions of type I (definite positive CT findings). The images (**a** CT; **b** SPECT; **c** fusion) were obtained from a 42-year-old man with PTC, who underwent total thyroidectomy 2 months ago. SPECT/CT demonstrated a RTT lesion with high-uptake and soft tissue nodule, adjacent to the thyroid right lamina (region IV). The images (**d** CT; **e** SPECT; **f** fusion) were obtained from a 15-year-old woman with FTC, who underwent total thyroidectomy 2 months ago. SPECT/CT revealed a RTT lesion high-uptake and soft tissue nodule, adjacent to the left side of tracheal cartilage (region I)

**Fig. 3:**
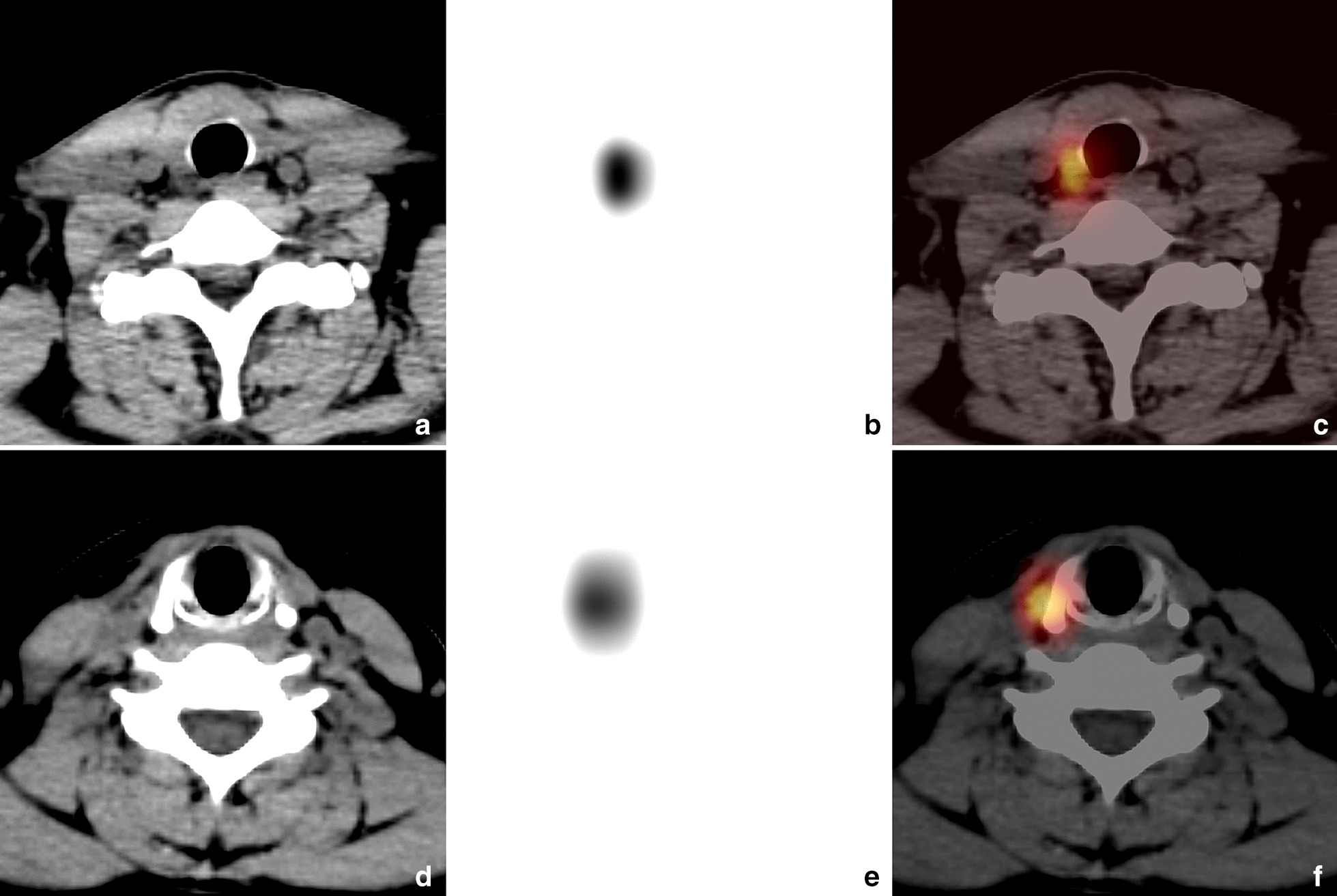
RTT lesions of type II (suspected positive CT findings). The images (**a** CT; **b** SPECT; **c** fusion) were obtained from a 38-year-old man with PTC, who underwent total thyroidectomy 3 months ago. SPECT/CT demonstrated a RTT lesion with high-uptake and suspected thickened soft tissue, adjacent to the right side of tracheal cartilage (region I). The images (**d** CT; **e** SPECT; **f** fusion) were obtained from a 31-year-old woman with PTC, who underwent total thyroidectomy 2 months ago. SPECT/CT revealed a RTT lesion with high-uptake and blurry patchy shadow, adjacent to the thyroid right lamina (region IV)

A total of 52 patients with DTC were included in this study, which were demonstrated 84 abnormal uptake foci on neck by ^131^I-WBS. SPECT/CT revealed a total of 106 foci on neck, among which 75.5%(80/106) were diagnosed as RTT, 20.8%(22/106) as thyroglossal tract and 3.8%(4/106) as metastatic lymph node. According to the CT findings, these 80 lesions of RTT were classified to type I of 10 (Fig. 2), type II of 21(Fig. 3) and type II of 49. Finally, 47 patients were confirmed the presence of RTT. The incidence rate of RTT was 90.4%(47/52).

### Location and SPECT/CT features of RTT

The detail locations and SPECT/CT findings of these 80 RTT lesions were shown in Table 2. Of these, most lesions were found with the features: region I (46.3%, 37/80) and region IV (21.3%, 17/30), high-uptake (57.5%. 46/80,) and moderate-uptake (30.0%, 24/80) and type III (61.3%, 49/80) and type II (26.3%, 21/80).

**Table 2:** Location and SPECT/CT features of RTT

Region	Number	SPECT uptake level			CT finding		
		Low-uptake	Moderate-uptake	High-uptake	Type I	Type II	Type III
I	37	5	8	24	4	12	21
II	11	2	3	6	0	4	7
III	10	1	5	4	1	2	7
IV	17	2	5	10	5	3	9
V	3	0	2	1	0	0	3
VI	2	0	1	1	0	0	2
Total	80	10	24	46	10	21	49

### The clinical data of patients with RTT

Based on the CT findings, 47 patients with RTT lesions were classified into positive group 29 (61.7%) and negative group 18 (38.3%). The correlation between various clinical data and groups was detailed shown in Table 3. By chi-square test, the data of primary thyroid tumor (*P* = 0.029) and T stage (*P* = 0.000) had the significant impact on the CT findings. However, other data (Age, Gender, N stage, M stage, Operation mode and Pathology) was not the effective risk factors.

**Table 3:** The correlation between various clinical data and groups

Data	CT findings		Total	*χ*^*2*^	*P*
	Positive group	Negative group			
Age					
≥ 55	2	4	6	0.072	0.789
< 55	16	25	41		
Gender					
Male	9	16	25	0.119	0.730
Female	9	13	22		
Pathology					
PTC	18	27	45	1.297	0.255
FTC	0	2	2		
Operation mode					
Mode 1–2	3	3	6	0.399	0.528
Mode 3	15	26	41		
Primary thyroid tumor					
Unilateral	15	8	23	4.778	0.029
Bilateral	8	16	24		
T stage					
T1	13	8	21	26.413	0.000
T2	10	7	17		
T3–4	2	7	9		
N stage					
N0	2–1	0	1	1.646	0.199
N1	21–17	29	46		
M stage					
M0	18	27	45	1.297	0.255
M1	0	2	2		

## Discussion

Total thyroidectomy is the first choice for the treatment of DTC, but RTT is usually existed in post-thyroidectomy patients. The results of this study indicated that the incidence rate of RTT was as high as 90.4%(47/52). Radioactive ^131^I therapy is routinely recommended in DTC patients after total thyroidectomy, to ablate postoperative RTT and treat microscopic residual tumor foci [11–13]. The dose of ^131^I ablation is closely related to the number and distribution of RTT. Accurate estimation of RTT before radioiodine therapy is essential to promote the individualized treatment [14]. In addition, RTT can also increase the risk of recurrence or metastasis of DTC, which may reduce the survival of patients. Therefore, in order to improve the comprehensive prognosis of DTC patients, it is very important to precisely detect RTT and minimize RTT.

At present, the alternative image evaluation methods for RTT include CT, ultrasound, techetium-99 m pertechnetate (^99m^TcO_4_^−^) planar scintigraphy, ^131^I-WBS (whole-body scan) and ^131^I-SPECT/CT. Ultrasound is a convenient method, which can clearly measure the shape and size of RTT. Lee SJ [15] reported that ultrasound was not as accurate as CT in assessing the volume of RTT. Planar scintigraphy can provide the information of iodine uptake. Tsai CJ [16] reported that ^131^I-WBS detected 206 lesions of RTT, while ^99m^TcO_4_^−^ planar scintigraphy revealed only 122 (59%) lesions. ^131^I is characterized by high sensitivity, which is conventionally performed as WBS in anterior and posterior views for the evaluation of RTT and metastatic lesions. ^131^I-SPECT/CT provides the precise localization of uptake foci, which helps to differentiate RTT in common locations from metastatic lymph nodes, as well as the identification of ectopic thyroid tissue, such as thyroglossal canal, root of tongue, etc. Cheng X [17] conducted a retrospective analysis of 73 DTC patients, and found that ^131^I-WBS made definite diagnosis only in 44(60.3%) patients, while ^131^I-SPECT/CT made it in 71(97.3%) patients. Spanu A [18] reported that 116 uptake foci from 52 patients were revealed by ^131^I-WBS, while a total of 158 foci from 59 patients were detected by ^131^I-SPECT/CT. Therefore, compared with ^131^I-WBS, ^131^I-SPECT/CT can significantly improve the diagnostic accuracy and detection rate of lesions [18, 19].

Although ^131^I SPECT/CT has been widely used for the evaluationg of RTT after thyroidectomy, while the distribution characteristics of RTT have not been systematically summarized. Avoiding the occurrence of RTT mainly depends on the careful clearance of surgeons, while the investigation on the distribution characteristics and risk factors of RTT will undoubtedly provide some guidance for improving the quality of surgery. In view of the lack of relevant literature, the present study originally divided the locations of RTT into six regions (I–VI). A total of 80 lesions of RTT were revealed by neck SPECT/CT, most of which were located in region I (46.3%, 37/80), followed by region IV (21.3%, 17/80). This results suggested that RTT lesions are more associated with trachea cartilage and lamina of thyroid cartilage. Its detail mechanism is not clear and need to be further explored. It may be related to the anatomical characteristics of thyroid. The slender tip of thyroid lateral lobe adjacent the lamina of thyroid cartilage makes it challenging for the surgeon to completely remove all thyroid tissue in that location. The long-term compression of tumor makes it necessary to reserve some soft tissues around the tracheal cartilage to avoid its collapse due to the loss of support. The intimae relationship between the RLN and the inferior thyroid artery (ITA) or Zuckerkandl tubercle (ZT) makes some thyroid tissue may be retained [20–24]. In addition, other generally accepted views on RTT generation include: (1) thyroid tissue left attached to a parathyroid gland in order to preserve its vascularity [25]; (2) thyroid tissue at the superior portion of the pyramidal lobe of the thyroid; (3) the defect of upper respiratory function and structure should be minimized in DTC resection.

This study demonstrated that most RTT (87.5%, 70/80, type II–III) were negative or suspected positive on CT, which could be interpreted as intraoperative invisible lesions and difficult to be detected and removed by surgeons. However, the remaining (12.5%, 10/80) RTT were revealed definite abnormalities on CT, most (50.0%) of them located in regions IV. These RTT lesions should be visible and detectable by naked eye during operation, which can be removed surgically. This means that the incidence of RTT may be reduced by about 10%, as long as the surgeons strengthen the careful removal of the correct regions, especially the region adjacent to lamina of thyroid cartilage. Moreover, this study also displayed that patients with DTC involving bilateral thyroid tissue or in T3–4 stage were more likely to have RTT with CT abnormalities after surgery. It suggests that surgeons should pay more attention to the careful removal when the primary thyroid tumor involving bilateral or T4 stage. Therefore, this study maybe provide a new idea and clear guidance for surgeons for the careful surgical removal.

## Conclusions

In summary, RTT has certain characteristic distribution and appearances on SPECT/CT. The definite positive CT findings can be noted in 12.50% of RTT, 50.0% of which were located adjacent to lamina of thyroid cartilage. It suggests that surgeons should strengthen the careful removal of thyroid tissue in this region, especially primary thyroid tumor involving bilateral and T4 stage. This study can provide a certain value for the improvement of thyroidectomy quality in DTC patients.

## Data Availability

Data and materials during the current study are available from the corresponding author upon reasonable request.
